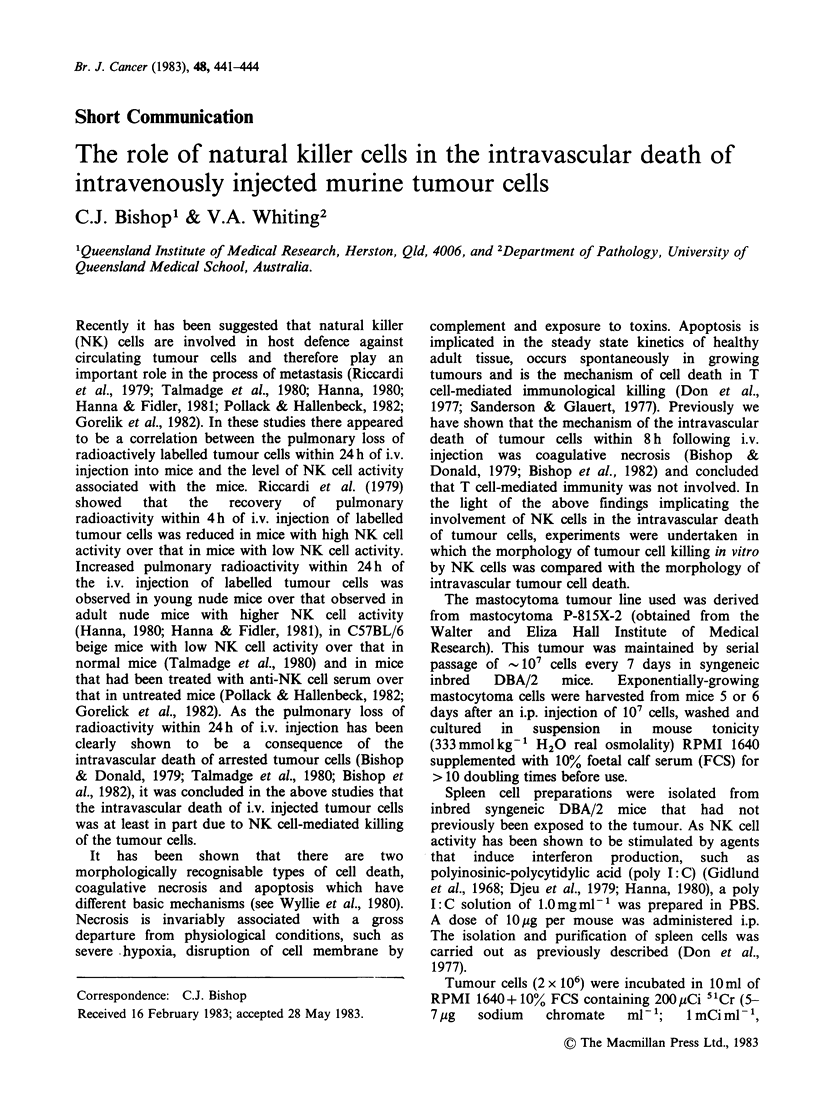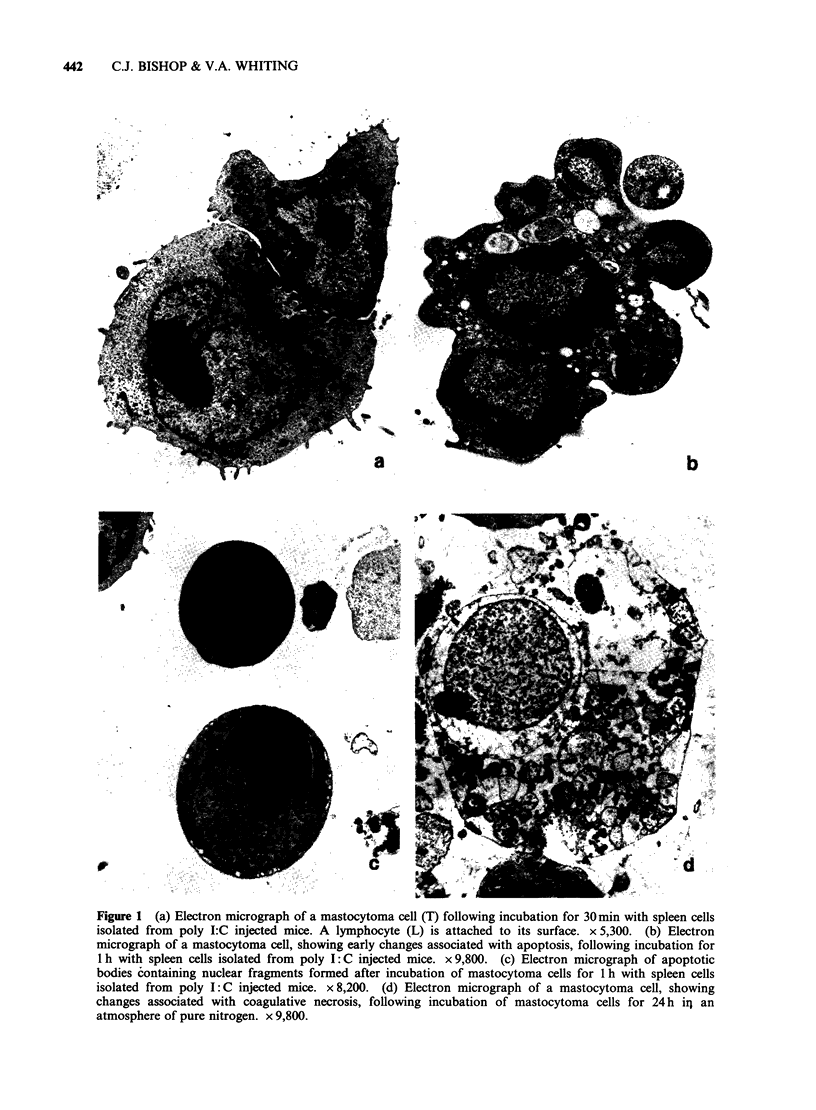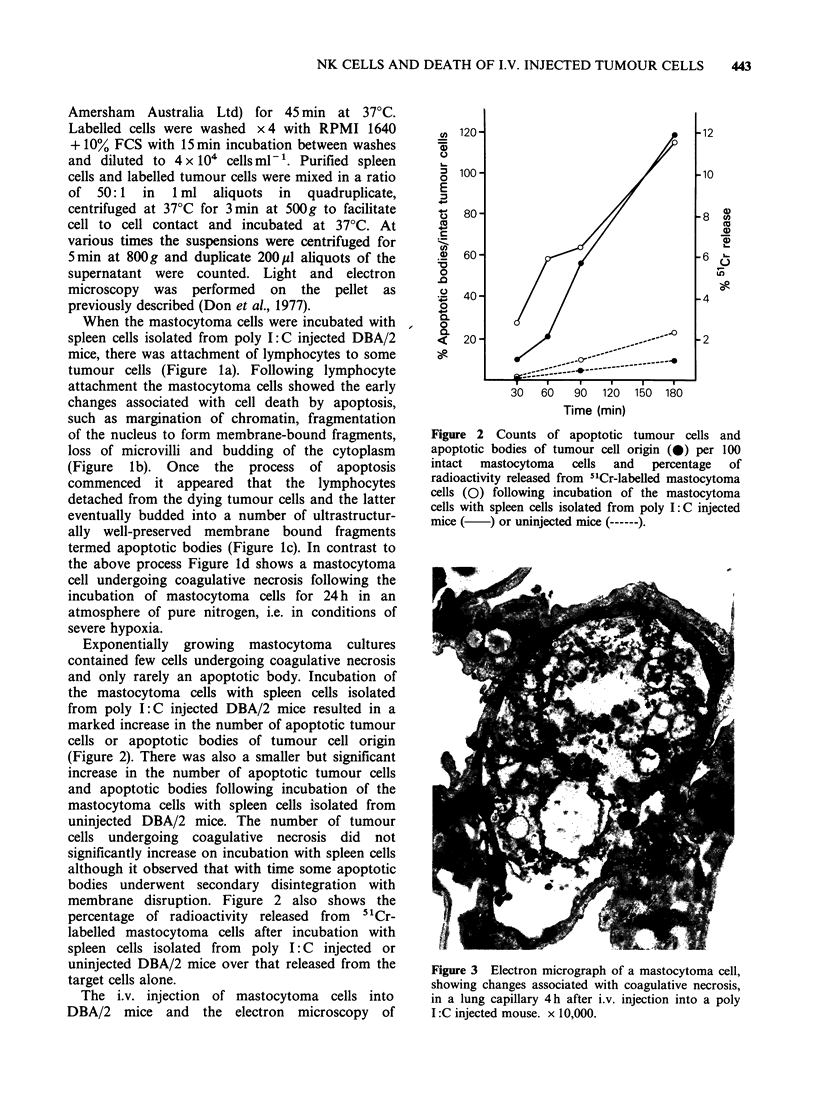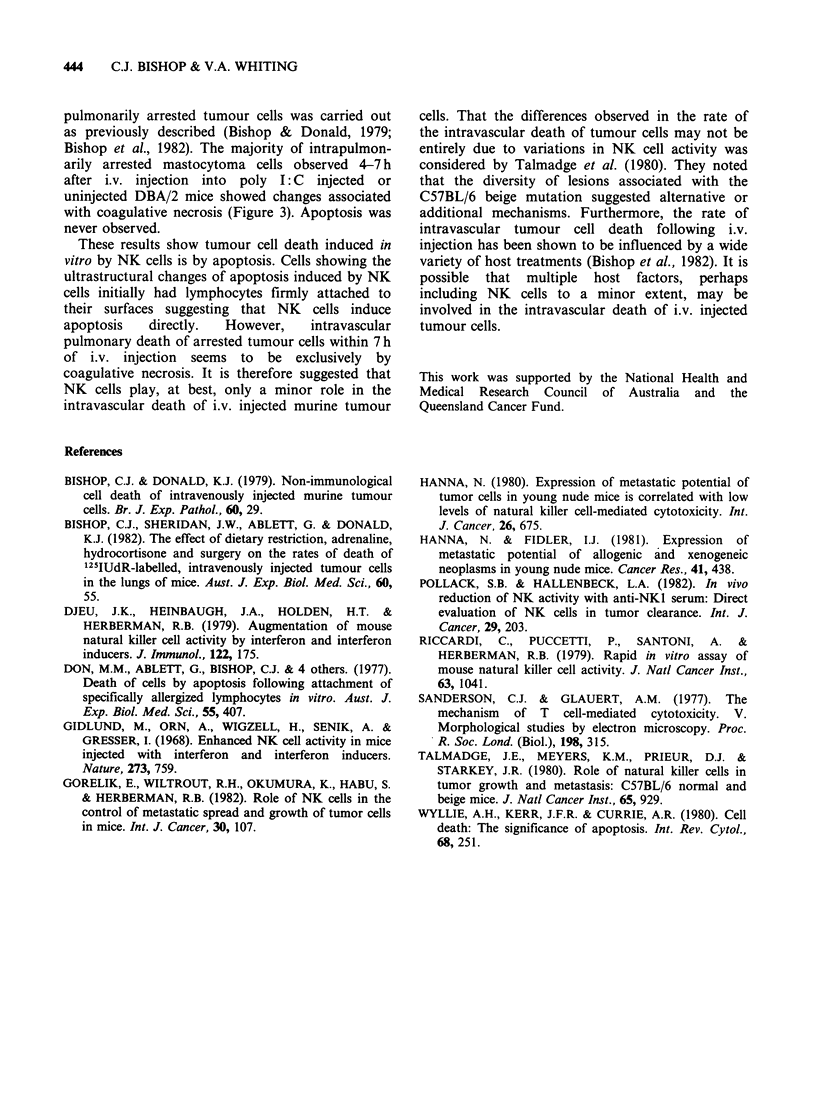# The role of natural killer cells in the intravascular death of intravenously injected murine tumour cells.

**DOI:** 10.1038/bjc.1983.211

**Published:** 1983-09

**Authors:** C. J. Bishop, V. A. Whiting

## Abstract

**Images:**


*Br. J. Cancer* (1983), **48**, 441–444

### Short Communication

### The role of natural killer cells in the intravascular death of intravenously injected murine tumour cells

C.J. Bishop[1] & V.A. Whiting[2]

[1]*Queensland Institute of Medical Research, Herston, Qld, 4006, and* [2]*Department of Pathology, University of Queensland Medical School, Australia.*

Recently it has been suggested that natural killer (NK) cells are involved in host defence against circulating tumour cells and therefore play an important role in the process of metastasis (Riccardi *et al.*, 1979; Talmadge *et al.*, 1980; Hanna, 1980; Hanna & Fidler, 1981; Pollack & Hallenbeck, 1982; Gorelik *et al.*, 1982). In these studies there appeared to be a correlation between the pulmonary loss of radioactively labelled tumour cells within 24 h of i.v. injection into mice and the level of NK cell activity associated with the mice. Riccardi *et al.* (1979) showed that the recovery of pulmonary radioactivity within 4 h of i.v. injection of labelled tumour cells was reduced in mice with high NK cell activity over that in mice with low NK cell activity. Increased pulmonary radioactivity within 24 h of the i.v. injection of labelled tumour cells was observed in young nude mice over that observed in adult nude mice with higher NK cell activity (Hanna, 1980; Hanna & Fidler, 1981), in C57BL/6 beige mice with low NK cell activity over that in normal mice (Talmadge *et al.*, 1980) and in mice that had been treated with anti-NK cell serum over that in untreated mice (Pollack & Hallenbeck, 1982; Gorelick *et al.*, 1982). As the pulmonary loss of radioactivity within 24 h of i.v. injection has been clearly shown to be a consequence of the intravascular death of arrested tumour cells (Bishop & Donald, 1979; Talmadge *et al.*, 1980; Bishop *et al.*, 1982), it was concluded in the above studies that the intravascular death of i.v. injected tumour cells was at least in part due to NK cell-mediated killing of the tumour cells.

It has been shown that there are two morphologically recognisable types of cell death, coagulative necrosis and apoptosis which have different basic mechanisms (see Wyllie *et al.*, 1980). Necrosis is invariably associated with a gross departure from physiological conditions, such as severe hypoxia, disruption of cell membrane by complement and exposure to toxins. Apoptosis is implicated in the steady state kinetics of healthy adult tissue, occurs spontaneously in growing tumours and is the mechanism of cell death in T cell-mediated immunological killing (Don *et al.*, 1977; Sanderson & Glauert, 1977). Previously we have shown that the mechanism of the intravascular death of tumour cells within 8 h following i.v. injection was coagulative necrosis (Bishop & Donald, 1979; Bishop *et al.*, 1982) and concluded that T cell-mediated immunity was not involved. In the light of the above findings implicating the involvement of NK cells in the intravascular death of tumour cells, experiments were undertaken in which the morphology of tumour cell killing *in vitro* by NK cells was compared with the morphology of intravascular tumour cell death.

The mastocytoma tumour line used was derived from mastocytoma P-815X-2 (obtained from the Walter and Eliza Hall Institute of Medical Research). This tumour was maintained by serial passage of $\sim 10^7$ cells every 7 days in syngeneic inbred DBA/2 mice. Exponentially-growing mastocytoma cells were harvested from mice 5 or 6 days after an i.p. injection of $10^7$ cells, washed and cultured in suspension in mouse tonicity (333 mmol kg$^{-1}$ $H_2O$ real osmolality) RPMI 1640 supplemented with 10% foetal calf serum (FCS) for $>10$ doubling times before use.

Spleen cell preparations were isolated from inbred syngeneic DBA/2 mice that had not previously been exposed to the tumour. As NK cell activity has been shown to be stimulated by agents that induce interferon production, such as polyinosinic-polycytidylic acid (poly I:C) (Gidlund *et al.*, 1968; Djeu *et al.*, 1979; Hanna, 1980), a poly I:C solution of 1.0 mg ml$^{-1}$ was prepared in PBS. A dose of 10 $\mu$g per mouse was administered i.p. The isolation and purification of spleen cells was carried out as previously described (Don *et al.*, 1977).

Tumour cells ($2 \times 10^6$) were incubated in 10 ml of RPMI 1640 + 10% FCS containing 200 $\mu$Ci $^{51}$Cr (5–7 $\mu$g sodium chromate ml$^{-1}$; 1 mCi ml$^{-1}$,

Correspondence: C.J. Bishop
Received 16 February 1983; accepted 28 May 1983.

© The Macmillan Press Ltd., 1983

**Figure 1** (a) Electron micrograph of a mastocytoma cell (T) following incubation for 30 min with spleen cells isolated from poly I:C injected mice. A lymphocyte (L) is attached to its surface. ×5,300. (b) Electron micrograph of a mastocytoma cell, showing early changes associated with apoptosis, following incubation for 1 h with spleen cells isolated from poly I:C injected mice. ×9,800. (c) Electron micrograph of apoptotic bodies containing nuclear fragments formed after incubation of mastocytoma cells for 1 h with spleen cells isolated from poly I:C injected mice. ×8,200. (d) Electron micrograph of a mastocytoma cell, showing changes associated with coagulative necrosis, following incubation of mastocytoma cells for 24 h in an atmosphere of pure nitrogen. ×9,800.

Amersham Australia Ltd) for 45 min at 37°C. Labelled cells were washed ×4 with RPMI 1640 +10% FCS with 15 min incubation between washes and diluted to $4 \times 10^4$ cells $ml^{-1}$. Purified spleen cells and labelled tumour cells were mixed in a ratio of 50:1 in 1 ml aliquots in quadruplicate, centrifuged at 37°C for 3 min at 500 *g* to facilitate cell to cell contact and incubated at 37°C. At various times the suspensions were centrifuged for 5 min at 800 *g* and duplicate 200 μl aliquots of the supernatant were counted. Light and electron microscopy was performed on the pellet as previously described (Don *et al.*, 1977).

When the mastocytoma cells were incubated with spleen cells isolated from poly I:C injected DBA/2 mice, there was attachment of lymphocytes to some tumour cells (Figure 1a). Following lymphocyte attachment the mastocytoma cells showed the early changes associated with cell death by apoptosis, such as margination of chromatin, fragmentation of the nucleus to form membrane-bound fragments, loss of microvilli and budding of the cytoplasm (Figure 1b). Once the process of apoptosis commenced it appeared that the lymphocytes detached from the dying tumour cells and the latter eventually budded into a number of ultrastructurally well-preserved membrane bound fragments termed apoptotic bodies (Figure 1c). In contrast to the above process Figure 1d shows a mastocytoma cell undergoing coagulative necrosis following the incubation of mastocytoma cells for 24 h in an atmosphere of pure nitrogen, i.e. in conditions of severe hypoxia.

Exponentially growing mastocytoma cultures contained few cells undergoing coagulative necrosis and only rarely an apoptotic body. Incubation of the mastocytoma cells with spleen cells isolated from poly I:C injected DBA/2 mice resulted in a marked increase in the number of apoptotic tumour cells or apoptotic bodies of tumour cell origin (Figure 2). There was also a smaller but significant increase in the number of apoptotic tumour cells and apoptotic bodies following incubation of the mastocytoma cells with spleen cells isolated from uninjected DBA/2 mice. The number of tumour cells undergoing coagulative necrosis did not significantly increase on incubation with spleen cells although it observed that with time some apoptotic bodies underwent secondary disintegration with membrane disruption. Figure 2 also shows the percentage of radioactivity released from $^{51}Cr$-labelled mastocytoma cells after incubation with spleen cells isolated from poly I:C injected or uninjected DBA/2 mice over that released from the target cells alone.

The i.v. injection of mastocytoma cells into DBA/2 mice and the electron microscopy of

**Figure 2** Counts of apoptotic tumour cells and apoptotic bodies of tumour cell origin (●) per 100 intact mastocytoma cells and percentage of radioactivity released from $^{51}Cr$-labelled mastocytoma cells (○) following incubation of the mastocytoma cells with spleen cells isolated from poly I:C injected mice (——) or uninjected mice (------).

**Figure 3** Electron micrograph of a mastocytoma cell, showing changes associated with coagulative necrosis, in a lung capillary 4 h after i.v. injection into a poly I:C injected mouse. ×10,000.

pulmonarily arrested tumour cells was carried out as previously described (Bishop & Donald, 1979; Bishop *et al.*, 1982). The majority of intrapulmonarily arrested mastocytoma cells observed 4–7 h after i.v. injection into poly I:C injected or uninjected DBA/2 mice showed changes associated with coagulative necrosis (Figure 3). Apoptosis was never observed.

These results show tumour cell death induced *in vitro* by NK cells is by apoptosis. Cells showing the ultrastructural changes of apoptosis induced by NK cells initially had lymphocytes firmly attached to their surfaces suggesting that NK cells induce apoptosis directly. However, intravascular pulmonary death of arrested tumour cells within 7 h of i.v. injection seems to be exclusively by coagulative necrosis. It is therefore suggested that NK cells play, at best, only a minor role in the intravascular death of i.v. injected murine tumour cells. That the differences observed in the rate of the intravascular death of tumour cells may not be entirely due to variations in NK cell activity was considered by Talmadge *et al.* (1980). They noted that the diversity of lesions associated with the C57BL/6 beige mutation suggested alternative or additional mechanisms. Furthermore, the rate of intravascular tumour cell death following i.v. injection has been shown to be influenced by a wide variety of host treatments (Bishop *et al.*, 1982). It is possible that multiple host factors, perhaps including NK cells to a minor extent, may be involved in the intravascular death of i.v. injected tumour cells.

This work was supported by the National Health and Medical Research Council of Australia and the Queensland Cancer Fund.

### References

BISHOP, C.J. & DONALD, K.J. (1979). Non-immunological cell death of intravenously injected murine tumour cells. *Br. J. Exp. Pathol.*, **60**, 29.

BISHOP, C.J., SHERIDAN, J.W., ABLETT, G. & DONALD, K.J. (1982). The effect of dietary restriction, adrenaline, hydrocortisone and surgery on the rates of death of $^{125}$IUdR-labelled, intravenously injected tumour cells in the lungs of mice. *Aust. J. Exp. Biol. Med. Sci.*, **60**, 55.

DJEU, J.K., HEINBAUGH, J.A., HOLDEN, H.T. & HERBERMAN, R.B. (1979). Augmentation of mouse natural killer cell activity by interferon and interferon inducers. *J. Immunol.*, **122**, 175.

DON, M.M., ABLETT, G., BISHOP, C.J. & 4 others. (1977). Death of cells by apoptosis following attachment of specifically allergized lymphocytes *in vitro*. *Aust. J. Exp. Biol. Med. Sci.*, **55**, 407.

GIDLUND, M., ORN, A., WIGZELL, H., SENIK, A. & GRESSER, I. (1968). Enhanced NK cell activity in mice injected with interferon and interferon inducers. *Nature*, **273**, 759.

GORELIK, E., WILTROUT, R.H., OKUMURA, K., HABU, S. & HERBERMAN, R.B. (1982). Role of NK cells in the control of metastatic spread and growth of tumor cells in mice. *Int. J. Cancer*, **30**, 107.

HANNA, N. (1980). Expression of metastatic potential of tumor cells in young nude mice is correlated with low levels of natural killer cell-mediated cytotoxicity. *Int. J. Cancer*, **26**, 675.

HANNA, N. & FIDLER, I.J. (1981). Expression of metastatic potential of allogenic and xenogeneic neoplasms in young nude mice. *Cancer Res.*, **41**, 438.

POLLACK, S.B. & HALLENBECK, L.A. (1982). *In vivo* reduction of NK activity with anti-NK1 serum: Direct evaluation of NK cells in tumor clearance. *Int. J. Cancer*, **29**, 203.

RICCARDI, C., PUCCETTI, P., SANTONI, A. & HERBERMAN, R.B. (1979). Rapid *in vitro* assay of mouse natural killer cell activity. *J. Natl Cancer Inst.*, **63**, 1041.

SANDERSON, C.J. & GLAUERT, A.M. (1977). The mechanism of T cell-mediated cytotoxicity. V. Morphological studies by electron microscopy. *Proc. R. Soc. Lond.* (Biol.), **198**, 315.

TALMADGE, J.E., MEYERS, K.M., PRIEUR, D.J. & STARKEY, J.R. (1980). Role of natural killer cells in tumor growth and metastasis: C57BL/6 normal and beige mice. *J. Natl Cancer Inst.*, **65**, 929.

WYLLIE, A.H., KERR, J.F.R. & CURRIE, A.R. (1980). Cell death: The significance of apoptosis. *Int. Rev. Cytol.*, **68**, 251.